# Supplementary material for: NF-κB inhibition in keratinocytes causes RIPK1-mediated necroptosis and skin inflammation
Source: Life Sci Alliance. 2021 Apr 15;4(6):e202000956. doi: 10.26508/lsa.202000956 (PMC8091601; doi:10.26508/lsa.202000956)
Supplement: Supplementary file 3 [file LSA-2020-00956_TableS1.docx]

**Table S1: IKK2^E-KO^ FADD^E-KO^ *Ripk3^-/-^* mice phenotype**

| **Mouse no.** | **Sacrifice Age (Days)** | **Macroscopic Observation** |
| --- | --- | --- |
| 1 | 149 | Lesion free |
| 2 | 146 | Lesion free |
| 3 | 134 | Lesion free |
| 4 | 149 | Lesion free |
| 5 | 149 | Lesion free |
| 6 | 149 | Lesion free |
| 7 | 145 | Lesion free |
| 8 | 182 | Lesion free |
| 9 | 224 | Lesion free |
| 10 | 224 | Lesion free |
| 11 | 221 | Lesion free |
| 12 | 323 | Lesion free |
| 13 | 328 | Lesion free |
| 14 | 384 | Lesion free |
| 15 | 384 | Lesion free |
| 16 | 333 | Lesion free |
| 17 | 333 | Lesion free |
| 18 | 333 | Lesion free |
| 19 | 333 | Lesion free |
| 20 | 333 | Mild lesion on the neck |
| 21 | 330 | Lesion free |
| 22 | 385 | Lesion free |
