# Supplementary material for: NF-κB inhibition in keratinocytes causes RIPK1-mediated necroptosis and skin inflammation
Source: Life Sci Alliance. 2021 Apr 15;4(6):e202000956. doi: 10.26508/lsa.202000956 (PMC8091601; doi:10.26508/lsa.202000956)
Supplement: Supplementary file 5 [file LSA-2020-00956_TableS2.docx]

**Table S2A: IKK2^E-KO^ *Ripk3^-/-^* mice phenotype**

| **Mouse no.** | **Sacrifice Age (Days)** | **Macroscopic Observation** |
| --- | --- | --- |
| 1 | 63 | Lesion free |
| 2 | 56 | Lesion free |
| 3 | 56 | Lesions on the ventral neck |
| 4 | 63 | Lesion free |
| 5 | 42 | Lesion free |
| 6 | 77 | Very mild lesions on the belly |
| 7 | 77 | Lesion free |
| 8 | 77 | Lesions on the belly |
| 9 | 77 | Mild lesions on the back, head and belly |
| 10 | 98 | Lesions on the belly and back |
| 11 | 79 | Lesion free |
| 12 | 79 | Lesions on the belly |
| 13 | 79 | Lesions on the belly |
| 14 | 78 | Lesions on the belly |
| 15 | 78 | Lesions on the belly |
| 16 | 78 | Lesions on the belly |
