# Supplementary material for: NF-κB inhibition in keratinocytes causes RIPK1-mediated necroptosis and skin inflammation
Source: Life Sci Alliance. 2021 Apr 15;4(6):e202000956. doi: 10.26508/lsa.202000956 (PMC8091601; doi:10.26508/lsa.202000956)
Supplement: Supplementary file 6 [file LSA-2020-00956_TableS3.docx]

**Table S2B: IKK2^E-KO^ RIPK3^E-KO^ mice phenotype**

| **Mouse no.** | **Sacrifice Age (Days)** | **Macroscopic Observation** |
| --- | --- | --- |
| 1 | 70 | Focal lesion on the back |
| 2 | 113 | Lesions on the belly |
| 3 | 151 | Mild focal lesion on the back |
| 4 | 105 | Focal lesions on the belly |
| 5 | 58 | Lesion free |
| 6 | 62 | Very mild focal lesion behind the ear |
| 7 | 62 | Lesion free |
| 8 | 107 | Very mild focal lesion on the neck |
| 9 | 83 | Lesions on the belly and back |
| 10 | 99 | Very mild focal lesion near ventral neck side |
| 11 | 99 | Mild focal lesions on the back |
| 12 | 147 | Lesions on the back and hind leg side |
