# Supplementary material for: NF-κB inhibition in keratinocytes causes RIPK1-mediated necroptosis and skin inflammation
Source: Life Sci Alliance. 2021 Apr 15;4(6):e202000956. doi: 10.26508/lsa.202000956 (PMC8091601; doi:10.26508/lsa.202000956)
Supplement: Supplementary file 8 [file LSA-2020-00956_TableS4.docx]

**Table S3A: IKK2^E-KO^ *Mlkl*^-/-^ mice phenotype**

| **Mouse no.** | **Sacrifice Age (Days)** | **Macroscopic Observation** |
| --- | --- | --- |
| 1 | 96 | Lesions on the lower belly |
| 2 | 103 | Lesions on the lower belly |
| 3 | 98 | Mild lesions on the belly |
| 4 | 99 | Lesions on the belly, back and sides |
| 5 | 66 | Mild lesions on the belly |
| 6 | 91 | Mild lesions on the belly and back |

**Table S3B: IKK2^E-KO^ *Ripk1*^D138N/D138N^ mice phenotype**
