# Supplementary material for: NF-κB inhibition in keratinocytes causes RIPK1-mediated necroptosis and skin inflammation
Source: Life Sci Alliance. 2021 Apr 15;4(6):e202000956. doi: 10.26508/lsa.202000956 (PMC8091601; doi:10.26508/lsa.202000956)
Supplement: Supplementary file 10 [file LSA-2020-00956_TableS5.docx]

| **Mouse no.** | **Sacrifice Age (Days)** | **Macroscopic Observation** |
| --- | --- | --- |
| 1 | 178 | Lesion free |
| 2 | 178 | Lesion free |
| 3 | 179 | Lesion free |
| 4 | 172 | Lesion free |
| 5 | 215 | Lesion free |
| 6 | 153 | Lesion free |
| 7 | 178 | Lesion free |
| 8 | 178 | Lesion free |
| 9 | 179 | Lesion free |
| 10 | 172 | Lesion free |
| 11 | 215 | Lesion free |
| 12 | 136 | Very mild focal lesion on the neck |
| 13 | 136 | Very mild focal lesion on the neck |
| 14 | 140 | Very mild lesions on the back |
| 15 | 258 | Very mild lesions on the back |
| 16 | 238 | Very mild lesions on the back |
| 17 | 380 | Lesion free |
| 18 | 384 | Lesion free |
| 19 | 281 | Lesion free |
| 20 | 410 | Lesion free |
