# Supplementary material for: NF-κB inhibition in keratinocytes causes RIPK1-mediated necroptosis and skin inflammation
Source: Life Sci Alliance. 2021 Apr 15;4(6):e202000956. doi: 10.26508/lsa.202000956 (PMC8091601; doi:10.26508/lsa.202000956)
Supplement: Supplementary file 11 [file LSA-2020-00956_TableS6.docx]

**Table S4A: RelA^E-KO^ cRel^E-KO^ *Mlkl^-/-^* mice phenotype**

| **Mouse no.** | **Sacrifice Age (Days)** | **Macroscopic Observation** |
| --- | --- | --- |
| 1 | 230 | Mild lesions on the snout and back |
| 2 | 130 | Very mild focal lesion on the back |
| 3 | 296 | Very mild focal lesion near ear |
| 4 | 278 | Very mild focal lesion near ear |
| 5 | 218 | Lesions on the neck and ear |
| 6 | 300 | Very mild focal lesion on the neck |

**Table S4B: RelA^E-KO^ c-Rel^E-KO^ *Ripk1*^D138N/D138N^ mice phenotype**
