# Supplementary material for: NF-κB inhibition in keratinocytes causes RIPK1-mediated necroptosis and skin inflammation
Source: Life Sci Alliance. 2021 Apr 15;4(6):e202000956. doi: 10.26508/lsa.202000956 (PMC8091601; doi:10.26508/lsa.202000956)
Supplement: Supplementary file 12 [file LSA-2020-00956_TableS7.docx]

| **Mouse no.** | **Sacrifice Age (Days)** | **Macroscopic Observation** |
| --- | --- | --- |
| 1 | 100 | Mild lesions on the back |
| 2 | 132 | Lesion on the neck |
| 3 | 135 | Lesion free |
| 4 | 135 | Lesion free |
| 5 | 135 | Lesion free |
| 6 | 135 | Lesion free |
| 7 | 207 | Lesion free |
| 8 | 180 | Lesion free |
| 9 | 258 | Lesion free |
| 10 | 172 | Lesion on the neck and back |
| 11 | 177 | Focal lesion on the back |
| 12 | 182 | Lesion free |
| 13 | 193 | Lesion on the ventral neck |
| 14 | 199 | Lesion on the neck |
| 15 | 202 | Lesions on the ventral and side neck |
| 16 | 214 | Lesions on the back |
| 17 | 216 | Focal lesion on the back |
| 18 | 243 | Lesion on the neck |
| 19 | 243 | Lesion on the ear |
| 20 | 258 | Lesions on the neck and back |
| 21 | 364 | Lesion free |
